# Composite Prediction Score to Interpret Bone Focal Uptake in Hormone-Sensitive Prostate Cancer Patients Imaged with [^18^F]PSMA-1007 PET/CT

**DOI:** 10.2967/jnumed.124.267751

**Published:** 2024-10

**Authors:** Matteo Bauckneht, Francesca D’Amico, Domenico Albano, Michele Balma, Camilla Cabrini, Francesco Dondi, Tania Di Raimondo, Virginia Liberini, Luca Sofia, Simona Peano, Mattia Riondato, Giuseppe Fornarini, Riccardo Laudicella, Luca Carmisciano, Egesta Lopci, Roberta Zanca, Marcello Rodari, Stefano Raffa, Maria Isabella Donegani, Daniela Dubois, Leonardo Peñuela, Cecilia Marini, Francesco Bertagna, Alberto Papaleo, Silvia Morbelli, Gianmario Sambuceti, Marta Ponzano, Alessio Signori

**Affiliations:** 1 Department of Health Sciences, University of Genova, Genova, Italy;; 2 Nuclear Medicine, IRCCS Ospedale Policlinico San Martino, Genova, Italy;; 3 Nuclear Medicine, ASST Spedali Civili di Brescia, Brescia, Italy;; 4 University of Brescia, Brescia, Italy;; 5 Nuclear Medicine, S. Croce e Carle Hospital, Cuneo, Italy;; 6 Medical Oncology 1, IRCCS Ospedale Policlinico San Martino, Genova, Italy;; 7 Nuclear Medicine, Department of Biomedical and Dental Sciences and Morpho-Functional Imaging, University of Messina, Messina, Italy;; 8 Department of Clinical and Experimental Medicine University of Pisa, Pisa, Italy;; 9 Nuclear Medicine, IRCCS, Humanitas Research Hospital, Rozzano, Italy;; 10 Institute of Molecular Bioimaging and Physiology, National Research Council, Milan, Italy;; 11 Nuclear Medicine, AOU Città della Salute e della Scienza, Turin, Italy; and; 12 Department of Medical Sciences, University of Turin, Turin, Italy

**Keywords:** prostate cancer, PET, prediction, prostate-specific membrane antigen, unspecific bone uptake

## Abstract

Unspecific bone uptake (UBU) related to [^18^F]PSMA-1007 PET/CT imaging represents a clinical challenge. We aimed to assess whether a combination of clinical, biochemical, and imaging parameters could predict skeletal metastases in patients with [^18^F]PSMA-1007 bone focal uptake, aiding in result interpretation. **Methods:** We retrospectively analyzed [^18^F]PSMA-1007 PET/CT performed in hormone-sensitive prostate cancer (PCa) patients at 3 tertiary-level cancer centers. A fourth center was involved in performing an external validation. For each, a volume of interest was drawn using a threshold method to extract SUV_max_, SUV_mean_, PSMA tumor volume, and total lesion PSMA. The same volume of interest was applied to CT images to calculate the mean Hounsfield units (HU_mean_) and maximum Hounsfield units. Clinical and laboratory data were collected from electronic medical records. A composite reference standard, including follow-up histopathology, biochemistry, and imaging data, was used to distinguish between PCa bone metastases and UBU. PET readers with less (*n* = 2) or more (*n* = 2) experience, masked to the reference standard, were asked to visually rate a subset of focal bone uptake (*n* = 178) as PCa metastases or not. **Results:** In total, 448 bone [^18^F]PSMA-1007 focal uptake specimens were identified in 267 PCa patients. Of the 448 uptake samples, 188 (41.9%) corresponded to PCa metastases. Ongoing androgen deprivation therapy at PET/CT (*P* < 0.001) with determination of SUV_max_ (*P* < 0.001) and HU_mean_ (*P* < 0.001) independently predicted bone metastases. A composite prediction score, the bone uptake metastatic probability (BUMP) score, achieving an area under the receiver-operating-characteristic curve (AUC) of 0.87, was validated through a 10-fold internal and external validation (*n* = 89 bone uptake, 51% metastatic; AUC, 0.92). The BUMP score’s AUC was significantly higher than that of HU_mean_ (AUC, 0.62) and remained high among lesions with HU_mean_ in the first tertile (AUC, 0.80). A decision-curve analysis showed a higher net benefit with the score. Compared with the visual assessment, the BUMP score provided added value in terms of specificity in less-experienced PET readers (88% vs. 54%, *P* < 0.001). **Conclusion:** The BUMP score accurately distinguished UBU from bone metastases in PCa patients with [^18^F]PSMA-1007 focal bone uptake at PET imaging, offering additional value compared with the simple assessment of the osteoblastic CT correlate. Its use could help clinicians interpret imaging results, particularly those with less experience, potentially reducing the risk of patient overstaging.

## Visual Abstract

**Figure:**
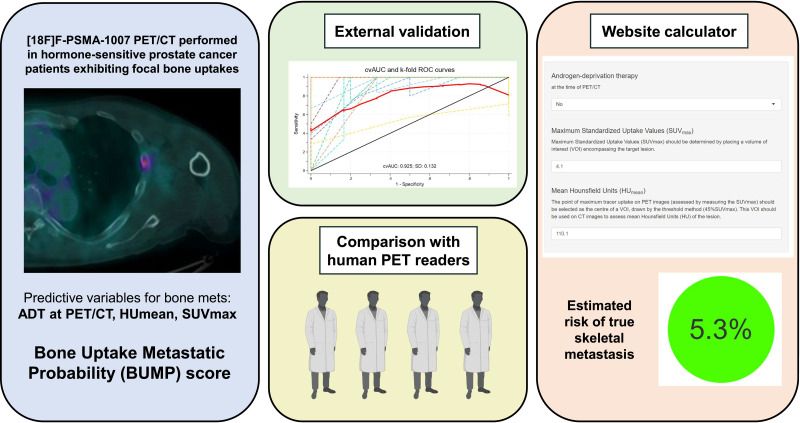

Prostate-specific membrane antigen (PSMA) PET/CT offers superior diagnostic accuracy over conventional imaging in prostate cancer (PCa) (1). After a substantial body of evidence was accumulated regarding [^68^Ga]Ga-PSMA-11 imaging (1*,*^2^), recent advances in logistics and the enhanced availability of [^18^F]PSMA-1007 have significantly broadened their use in PSMA PET imaging for PCa. Indeed, [^18^F]PSMA-1007 benefits from a prevalent nonurinary excretion, which may enhance the visualization of cancer sites in the pelvis (3*,*^4^). Nonetheless, its propensity for noncancerous bone tissue accumulation poses a challenge, often leading to ambiguous imaging results. Although the precise mechanism behind this phenomenon remains unclear (5–15), unspecific bone uptake (UBU) has been observed in as many as 72% of PCa patients (13), complicating the interpretation of scan results.

In clinical practice, the misinterpretation of UBU as metastatic lesions could result in disease overstaging, potentially causing missed opportunities for curative treatments or unnecessary therapy escalation (2*,*^16^–^18^). This issue underscores the need for accurate tools to differentiate between malignant and benign bone lesions in PCa patients undergoing [^18^F]PSMA-1007 PET/CT.

To address this challenge, we hypothesized that the likelihood of identifying bone focal uptake due to metastatic sites in [^18^F]PSMA-1007 PET/CT scans could be predicted by analyzing a comprehensive set of factors, including clinical history, biochemical markers, and the specific imaging characteristics of the bone uptake. We developed and validated a comprehensive prediction model to distinguish between UBU and bone metastases, thereby enhancing the diagnostic accuracy and potentially improving patient management strategies.

## MATERIALS AND METHODS

### Study Population

We conducted a retrospective analysis in a real-world setting on patients with histology-proven hormone-sensitive PCa who consecutively underwent [^18^F]PSMA-1007 PET/CT at 3 tertiary-level cancer centers between July 2020 and May 2023. A fourth center was involved in performing an external validation.

**FIGURE 1.**
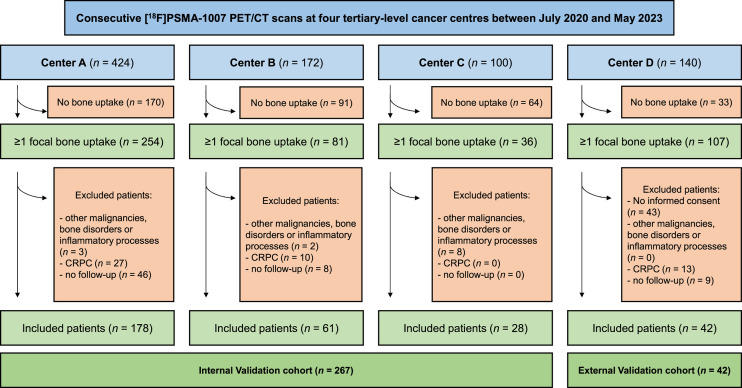
Patient inclusion flow chart shows patient selection. Center A: University of Genova; Center B: University of Brescia; Center C: S. Croce e Carle Hospital, Cuneo; Center D: Humanitas Research Hospital, Rozzano. CRPC = castration-resistant prostate cancer.

The study’s sole inclusion criterion was identifying the presence of at least 1 bone focal uptake, exhibiting greater intensity than the surrounding bone marrow, regardless of a corresponding CT correlate. Patients with synchronous malignancies, known bone disorders, or active inflammatory processes and castration-resistant PCa were excluded (the Discussion section provides details on this choice). The patient selection process is illustrated in Figure 1. The study adhered to the Declaration of Helsinki and was approved by the local ethics committee (code 5/2023, database identifier 12914). Written informed consent was provided by the enrolled patients.

### Imaging Procedures, Analyses, and Data Collection

PET/CT scans were acquired a median of 95 min (interquartile range, 90–117 min) after the administration of a median dose of 311 MBq (interquartile range, 277–342 MBq) of [^18^F]PSMA-1007, according to the current guidelines (19). All studies were performed using dedicated state-of-the-art Biograph Hi REZ 16 (Siemens Healthineers), Biograph MCT Flow (Siemens Healthineers), Discovery 690 and ST (GE Healthcare), and 3-dimensional time-of-flight Ingenuity TF PET/CT system (Philips) scanners. Regardless of the scanner used, CT images were obtained with the following acquisition parameters: 120 kV, 30–400 mA, and 0.984:1/39.37 pitch.

One experienced nuclear medicine physician at each center independently and retrospectively identified visually the presence or absence of [^18^F]PSMA-1007 focal bone uptake. If present, the number and regional site were recorded. Images were interpreted according to the E-PSMA standardized reporting guidelines (20). SUV_max_ was determined by placing a volume of interest encompassing the target lesion. The PSMA visual score (20) was also recorded. The point of maximum tracer uptake (assessed by measuring SUV_max_) was selected as the center of a volume of interest, drawn by the threshold method (45% SUV_max_ as previously described (21)) to calculate SUV_mean_, PSMA tumor volume, and total lesion PSMA. The same volume of interest was used on CT images to assess mean and maximum Hounsfield units (HU_mean_ and HU_max_, respectively). Clinical and laboratory data at the time of [^18^F]PSMA-1007 PET/CT were collected from the electronic medical records.

### Standard Reference Definition

Based on clinical, biochemical, and radiologic follow-up, a composite reference standard was used as previously described (22). The confirmation of bone metastases was based on changes in the size, the spontaneous disappearance, or the appearance of a CT correlate at imaging follow-up without any treatments or by a decrease in prostate-specific antigen of at least 50% after metastasis-directed therapy. For this study, lesions classified as uncertain were considered nonmetastatic. Histopathology was available for a subgroup of lesions and was considered the gold standard reference in these cases.

### Statistical Analysis, Composite Score Development, and Comparison with Visual Assessment

Patient characteristics were presented using absolute frequency and percentage for categoric variables and median and interquartile range for quantitative variables. Differences were considered statistically significant at a *P* value of less than 0.05. Uni- and multivariable logistic regression models were performed to identify independent predictors between each clinical, laboratory, or imaging parameter of bone metastases, as defined by the reference standard in a lesion-based analysis while considering intrapatient correlations. The results were expressed as odds ratios with 95% CIs. For the composite score development, using the bone uptake metastatic probability (BUMP) score, the variables included in the multivariable analyses were selected using stepwise selection across all factors with a *P* value of less than 0.10 in the univariable analysis, except for HU_max_ and initial prostate-specific antigen, because of multicollinearity issues, and the center was set as an offset variable. A Lasso model was then performed using cross-validation, and penalized coefficients were used to derive the score. We derived the cross-validated area under the receiver-operating-characteristic curve (cvAUC), the Brier score, and the calibration and decision curve plots to evaluate the score. The cvAUC and the Brier score were also measured in the external validation cohort. To explore the added value of the BUMP score to visual assessment, PET readers classified as having a low (<30 prior [^18^F]PSMA-1007 PET/CT reads, *n* = 2) or a high level of experience (>600 prior [^18^F]PSMA-1007 PET/CT reads, *n* = 2) (22), masked to reference standard, were asked to visually classify a subset of focal bone uptake (*n* = 178) as PCa bone metastases or not. The best BUMP score cutoff for the probability of bone metastasis was calculated with the Liu criterion (23). The BUMP score assessment was then compared with the visual assessments of PET readers in terms of specificity. Statistical analyses were conducted using Stata, version 18 (StataCorp LLC).

## RESULTS

### Internal Cohort

In total, 448 focal bone uptake sites of [^18^F]PSMA-1007 were identified in 267 patients with PCa at 3 centers (Fig. 1). In 10 cases, histopathology served as the reference standard, whereas a composite standard reference was applied for the remainder. According to the reference standard, 188 of these uptake sites (41.9%) were confirmed as PCa metastases. Among the remaining 260 uptake sites, 201 were classified as nonmetastatic (representing 44.9% of the total), whereas 59 were uncertain at follow-up (accounting for 13.2%). However, these uncertain uptake sites were subsequently considered nonmetastatic. Clinical characteristics and imaging data are summarized in Table 1.

**TABLE 1.** Clinical Characteristics and Imaging Data for Bone Uptake of [^18^F]PSMA-1007

	Training cohort (total *n* = 448)	External validation cohort (total *n* = 89)
Clinical setting		
Primary	255 (56.9)	24 (27.0)
Restaging	193 (43.1)	65 (73.0)
Initial PSA (ng/mL)	10.2 (6.3–22.9)	10.6 (5.6–227)
ISUP grade group		
1	25 (5.6)	16 (18.0)
2	99 (22.1)	6 (6.7)
3	114 (25.5)	17 (19.1)
4	112 (25.0)	10 (11.2)
5	66 (14.7)	40 (44.9)
Missing	32 (7.1)	0 (0.0)
PSA at PET/CT (ng/mL)	6.3 (1.5–16.4)	3.0 (0.52–17)
ADT at PET/CT		
No	343 (76.6)	63 (70.8)
Yes	104 (23.2)	26 (29.2)
Unknown	1 (0.2)	0 (0)
PSMA injected dose (MBq)	311 (277–342)	352 (337–362)
Uptake time (min)	95.0 (90.0–117.0)	73.0 (60.0–88.0)
Bone uptake site		
Ribs	145 (32.4)	27 (30.3)
Spine	120 (26.7)	17 (19.1)
Pelvis	132 (29.5)	24 (27.0)
Other sites	51 (11.4)	21 (23.6)
HU_mean_	192.4 (131–277.4)	157 (111–248)
HU_max_	663 (455–837)	412 (268–669)
SUV_max_	5.2 (4.0–9.7)	5.6 (4–12.7)
PSMA visual score	1.0 (1.0–2.0)	1.0 (1.0–3.0)
PSMA-TV (mL)	1.8 (1–3.3)	1.4 (0.7–3.1)
TL-PSMA	6.6 (3.8–14.4)	4.8 (2.4–14.4)

PSA = prostate-specific antigen; ISUP = International Society of Urological Pathology; PSMA-TV = PSMA tumor volume; TL-PSMA = total lesion PSMA.

Qualitative data are number and percentage. Continuous data are median and interquartile range.

### BUMP Score Determinants

**FIGURE 2.**
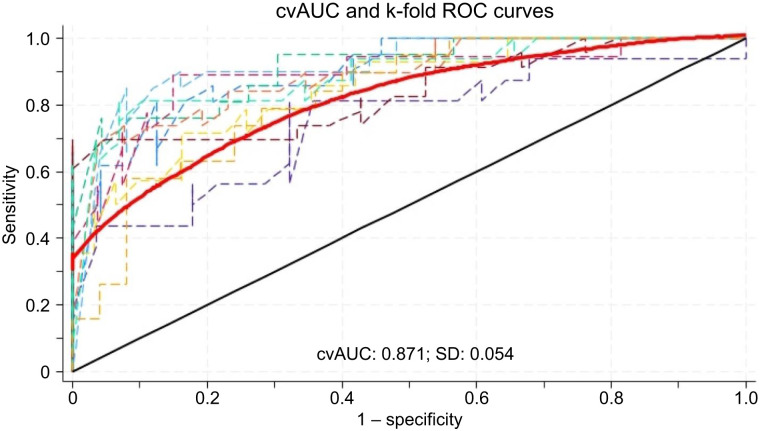
Model discrimination. Receiver-operating-characteristic (ROC) curve (red line) shows mean cvAUC, resulting after 10-fold cross-validation (dashed lines).

**FIGURE 3.**
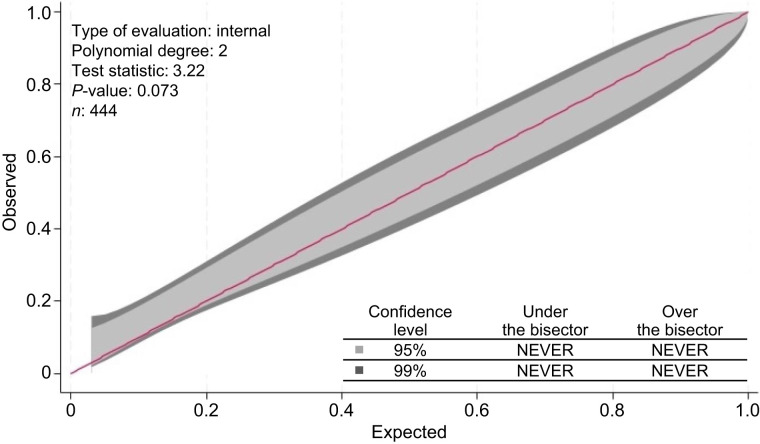
Internal calibration plot shows deviation from 45° line of perfect fit (red) at 95% CI (light gray) and 99% CI (dark gray).

At the univariate per-lesion analysis, PET/CT indication (primary staging vs. restaging), prostate-specific antigen levels at the time of initial diagnosis and at the time of PET/CT, initial International Society of Urological Pathology grade group, ongoing androgen-deprivation therapy (ADT) at the time of PET/CT, the site of bone tracer uptake, SUV_max_, total lesion PSMA, HU_mean_, and HU_max_ were associated with the presence of PCa metastases (Table 2). In the multivariate analysis, the ongoing ADT at PET/CT with determination of SUV_max_ and HU_mean_ resulted in independent predictors of bone metastases (Table 2). The score based on Lasso-penalized coefficients combining these parameters achieved an AUC of 0.8656. This AUC was validated through 10-fold internal cross-validation, obtaining a cvAUC of 0.8707 (95% CI, 0.8326–0.9030) (Fig. 2; Brier score, 0.1405). The hypothesis of the good calibration was not rejected (*P* = 0.073), and consistently, the 95% and 99% calibration belts encompassed the bisector over the whole range of the predicted probabilities, suggesting that the model’s internal calibration was acceptable (Fig. 3). A sensitivity analysis showed that the HU_mean_ cvAUC was significantly inferior to the BUMP score (AUC, 0.62 vs. 0.87). Similarly, the performance of the BUMP score remained high among lesions with HU_mean_ in the first tertile (*n* = 149; median HU_mean_, 110; interquartile range, 71–131), where the cvAUC was 0.806. When the coefficients from the predictors retained in the model was used (Table 3), the probability of a true metastasis was equal to the inverse of a logistic regression equation as follows:

$$\text{Individual}\;\text{probability}\;\text{of}\;\text{bone}\;\text{metastasis}=\frac{1}{\left(1+e^{-\text{BUMP}\;\text{score}}\right)},$$

where the BUMP score is calculated as

−4.660246 + 1.690656 (if ADT at PET) + 0.0043016× Umean + 0.317027 × SUVmax.

**TABLE 2.** Univariate and Multivariate Analyses Identifying Determinants of the BUMP Score

Parameter	Nonmetastatic (total *n* = 260)	Metastatic (total *n* = 188)	Univariate analyses		Multivariate analyses	
			OR	*P*	OR	*P*
Clinical setting						
Primary staging	167 (65.5)	88 (34.5)	1.00 (Ref)			
Restaging	93 (48.2)	100 (51.8)	2.04 (1.18–3.52)	0.010	—	NS
Initial PSA (ng/mL)	8.7 (6.0–16.5)	14.0 (7.7–33.0)	1.48 (1.16–1.88)	0.001	—	NS
ISUP grade group						
1	17 (68.0)	8 (32.0)	1.00 (Ref)			
2	77 (77.8)	22 (22.2)	0.61 (0.18–2.08)			
3	70 (61.4)	44 (38.6)	1.34 (0.41–4.32)			
4	53 (47.3)	59 (52.7)	2.37 (0.71–7.92)			
5	31 (47.0)	35 (53.0)	2.40 (0.70–8.26)	0.009	—	NS
Missing	12 (37.5)	20 (62.5)	—	—		
PSA at PET/CT (ng/mL)	6.2 (1.1–13.0)	6.8 (3.0–27.0)	1.19 (1.02–1.38)	0.027	—	NS
ADT at PET/CT						
No	230 (67.1)	113 (32.9)	1.00 (Ref)		1.00 (Ref)	
Yes	30 (28.8)	74 (71.2)	5.02 (2.62–9.63)	<0.001	4.06 (1.91–8.67)	<0.001
Unknown	0 (0)	1 (100)	—	—		
PSMA injected dose (MBq)	309.0 (277.0–339.5)	312.0 (281.0–343.0)	1.03 (0.98–1.08)	0.277		
Uptake time (min)	96.0 (90.0–116.0)	95.0 (90.0–117.0)	1.01 (0.88–1.16)	0.902		
Bone uptake site						
Ribs	104 (71.7)	41 (28.3)	1.00 (Ref)			
Spine	70 (58.3)	50 (41.7)	1.81 (1.19–2.76)			
Pelvis	66 (50.0)	66 (50.0)	2.54 (1.61–3.99)			
Other sites	20 (39.2)	31 (60.8)	3.93 (1.96–7.89)	<0.001	—	NS
HU_mean_	180.6 (118.3–247.0)	211.4 (150.9–320.5)	1.03 (1.01–1.05)	0.001	1.04 (1.02–1.06)	<0.001
HU_max_	592.0 (420.0–790.0)	744.5 (541.0–926.0)	1.02 (1.01–1.02)	<0.001	—	NS
SUV_max_	4.5 (3.7–5.6)	10.2 (5.2–21.9)	1.35 (1.25–1.46)	<0.001	1.39 (1.27–1.52)	<0.001
PSMA visual score	1.0 (1.0–1.0)	2.0 (1.0–3.0)	2.38 (1.79–3.17)	<0.001	—	NS
PSMA-TV (mL)*	1.8 (1.1–3.0)	1.8 (0.9–3.7)	1.00 (0.96–1.05)	0.947		
TL-PSMA*	5.0 (3.4–9.2)	11.1 (5.0–30.0)	1.02 (1.01–1.04)	0.012	—	NS

* Variables were evaluated independently in multivariate analysis to avoid collinearity instability.

OR = odds ratio; Ref = reference value; NS = not significant; PSA = prostate-specific antigen; ISUP = International Society of Urological Pathology; PSMA-TV = PSMA tumor volume; TL-PSMA = total lesion PSMA.

Qualitative data are number and percentage. Continuous data are median and interquartile range. OR data are number and 95% CI in parentheses.

**TABLE 3.** Penalized Coefficients After 10-Fold Cross-Validation Lasso Model

Parameter	Penalized regression coefficients
Intercept	−4.660246
ADT at time of PET/CT	1.690656
HU_mean_	0.0043016
SUV_max_	0.317027

A website featuring the BUMP score calculator is accessible at https://scorecalc.shinyapps.io/BUMP/.

**FIGURE 4.**
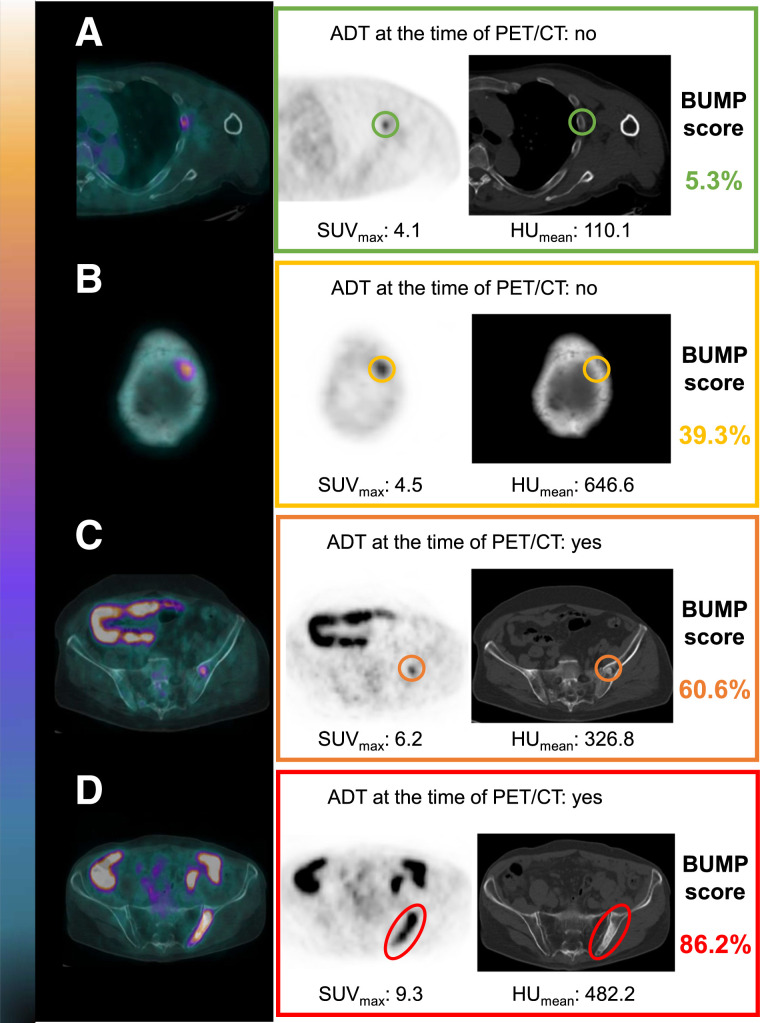
Emblematic examples of bone uptake of [^18^F]PSMA-1007 lead to varying probabilities of bone metastases when BUMP score is applied. According to BUMP score results, 4 cases with increasing probabilities of bone metastases are shown in panels top to bottom.

Figure 4 shows representative cases of bone uptake of [^18^F]PSMA-1007, resulting in divergent BUMP scores predicting low and high probabilities of bone metastases.

### External Validation Cohort

We then considered an external validation cohort at a fourth center (Fig. 1), including 89 bone uptake sites (51% metastatic) in 42 patients with PCa (Table 1). In the validation set, our score demonstrated excellent discrimination for BUMP with a cvAUC of 0.925 (95% CI, 0.748–0.929; Supplemental Fig. 1; supplemental materials are available at http://jnm.snmjournals.org) and a Brier score of 0.1507.

### Decision-Curve Analysis

**FIGURE 5.**
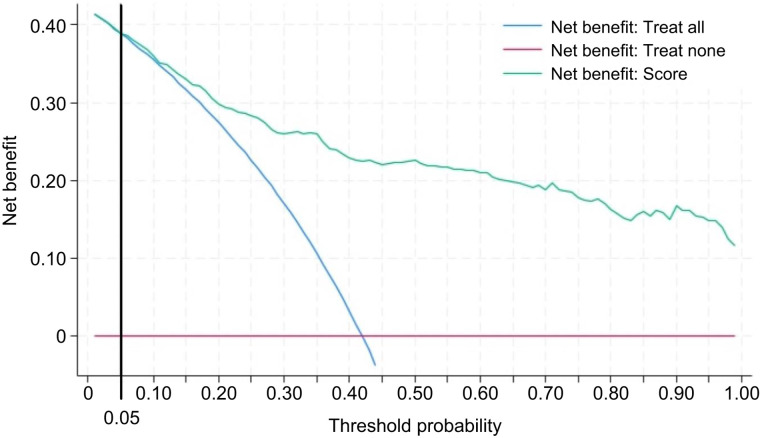
Decision-curve analysis demonstrates clinical net benefit against threshold probability associated with using BUMP score for interpreting focal bone uptake in hormone-sensitive PCa patients imaged with [^18^F]PSMA-1007 PET/CT.

The decision-curve analysis depicted in Figure 5 shows that, except for a small range of low threshold probabilities (range, 0–0.05), intervening on patients on the basis of the score leads to higher net benefit than intervening for all or none.

### Comparison Between the BUMP Score and PET Readers’ Visual Assessment

After the BUMP score was dichotomized (cutoff, 0.25), its specificity was superior to that of less-experienced readers (88% vs. 54%, *P* < 0.001), whereas it did not provide added value compared with results from highly experienced readers (88% vs. 94%, *P* < 0.05).

## DISCUSSION

Although [^18^F]PSMA-1007 significantly broadened the use of PET imaging in PCa, its propensity for bone focal uptake poses a clinical challenge. In this study, we developed and externally validated a composite prediction score with high accuracy for distinguishing between UBU and bone metastases in PCa patients with focal bone uptake. The BUMP score could assist clinicians in accurately interpreting imaging results, thereby reducing the risk of overstaging patients.

The determinants of the BUMP score, SUV_max_, HU_mean_, and ADT at the time of PET/CT, were selected on the basis of their significant association with the presence of bone metastases in multivariate analysis. SUV_max_ directly measures the intensity of radiotracer uptake, correlating with the level of PSMA expression activity. Its predictive power stems from its ability to quantify the intensity of PSMA expression in bone lesions, effectively distinguishing between aggressive metastatic disease and benign conditions with lower PSMA expression. HU_mean_, which indicates bone lesion density, provides insights into the lesion’s nature. Higher HU_mean_ values independently predict sclerotic bone metastases, likely because of a denser bone reaction surrounding metastatic lesions than benign conditions. In line with this, an accurate visual inspection of the CT correlate of focal bone tracer uptake remains the standard for image interpretation. However, bone density assessment alone might not be sufficient to provide a differential diagnosis between UBU and bone metastasis. Indeed, in our study, combining HU_mean_ with other variables provided additional value compared with using HU_mean_ alone. Furthermore, the BUMP score maintained high diagnostic accuracy even in lesions with lower HU_mean_ values, which are less sclerotic on visual inspection and, thus, the most challenging to differentiate in clinical practice. The observed predictive power of concurrent ADT administration during imaging may seem less intuitive. We hypothesize that the positive association indicates that patients undergoing ADT at the time of imaging are likely in more advanced disease stages, thus having a higher probability of true positive bone lesions. In a previous study, Ahmadi Bidakhvidi et al. (24) explored variables predicting the type and number of lesions detected by [^18^F]PSMA-1007 PET/CT in 175 patients with biochemical recurrence of PCa following primary therapy. Prior and ongoing ADT resulted in clinical predictors of bone lesions in univariable analyses, with prior ADT emerging as a significant independent predictor in multivariable analysis (24). However, their assessment was limited to biochemical and clinical parameters, excluding quantitative imaging characteristics and without the corroboration of histopathology or follow-up confirmation. Despite these considerations, the predictive value of this clinical characteristic underscores the importance for nuclear medicine physicians to integrate clinical parameters alongside imaging features in their [^18^F]PSMA-1007 PET/CT reports.

The decision-curve analysis offers valuable insights into the clinical utility of the BUMP score, supporting its application in clinical decision-making. Notably, there’s an exception for a narrow band of low-threshold probabilities (0%–5%). This finding underscores that using the BUMP score to guide clinical interventions proves to be more advantageous than the blanket strategy of intervening in all patients or refraining from interventions above the 5% threshold. This highlights the score’s significant practical value in enhancing patient care. Notably, this tool could be particularly advantageous for less-experienced PET readers as it may improve specificity, mitigating the risk of overstaging associated with the phenomenon of UBU. This observation aligns with previous literature, demonstrating that the propensity of [^18^F]PSMA-1007 for bone uptake does not increase the incidence of bone metastases diagnosed by experienced readers (14*,*^15^).

Some limitations warrant attention. The primary limitation of this retrospective analysis is the lack of histopathologic confirmation of most bone lesions. However, ethical considerations regarding additional pain and difficulties in performing PET/CT-guided biopsies would not justify a large cohort with biopsy-proven metastasis. Although an expert reader at each participating center visually identified focal bone uptake, the absence of a centralized review process may have introduced selection bias due to variability in imaging interpretation. Similarly, the retrospective study prevented interscanner cross-calibration, potentially resulting in low SUV reproducibility. These choices were made to develop a score adapted to real-world clinical practice. Relying on visual identification of focal bone uptake without setting quantitative uptake intensity thresholds was essential to avoid introducing selection bias due to SUV interscanner variability. By setting SUV_max_ as a continuous rather than a discrete variable, including the center as an offset variable in our multivariable model, and through the score’s external validation, we were able to lower the impact of interobserver and interscanner variability on the score’s clinical applicability. Consistent with findings from previous studies (6*,*^25^), we observed significant variability in UBU frequencies across centers. Again, this variability was accounted for by including the center as an offset variable in our multivariate model. Finally, we must acknowledge a potential source of heterogeneity in our cohort related to ongoing therapy, which may influence PSMA expression (26). However, the impact of this bias is somewhat mitigated since the study focused on patients with hormone-sensitive PCa. Indeed, we excluded patients with castration-resistant PCa, as treatments commonly administered in castration-resistant PCa, such as bone-targeting agents and palliative skeletal irradiation, could affect the tracer’s distribution and the morphology observed in CT images. We considered this decision not detrimental to the study’s relevance, as the clinical challenges posed by UBU are more significant in the early stages of the disease’s natural history.

## CONCLUSION

The BUMP score distinguished UBU from bone metastases in PCa patients with [^18^F]PSMA-1007 focal bone uptake. Its use could support clinicians approaching [^18^F]PSMA-1007 PET/CT interpretation, particularly if less experienced, thus potentially reducing the risk of patient overstaging.

## DISCLOSURE

This work was supported by the Italian Ministry of Health (Ricerca Corrente) granted to Matteo Bauckneht. Matteo Bauckneht reports personal fees from AAA and GE Healthcare. Egesta Lopci reports receiving a grant from the Italian Ministry of Health (Ministero della Salute). No other potential conflict of interest relevant to this article was reported.

## Notes

KEY POINTS **QUESTION:** Can combined clinical, biochemical, and imaging parameters predict bone metastases in patients showing bone focal uptake at [^18^F]PSMA-1007 PET/CT? **PERTINENT FINDINGS:** In a retrospective analysis of [^18^F]PSMA-1007 PET/CT scans from hormone-sensitive PCa patients, ongoing ADT, SUV_max_, and HU_mean_ independently predicted bone metastases. A composite prediction score (the BUMP score) demonstrated a cvAUC of 0.87, accurately distinguishing UBU from bone metastases. **IMPLICATIONS FOR PATIENT CARE:** The BUMP score’s application in clinical settings may assist clinicians in interpreting [^18^F]PSMA-1007 PET/CT imaging results, potentially reducing the risk of overstaging PCa.
